# A recombinant Der p 1-specific allergen-toxin demonstrates superior killing of allergen-reactive IgG^+^ hybridomas in comparison to its recombinant allergen-drug conjugate

**DOI:** 10.1093/immadv/ltac023

**Published:** 2022-12-06

**Authors:** A K Daramola, O A Akinrinmade, E A Fajemisin, K Naran, N Mthembu, S Hadebe, F Brombacher, A M Huysamen, O E Fadeyi, R Hunter, S Barth

**Affiliations:** South African Research Chair in Cancer Biotechnology, Department of Integrative Biomedical Sciences, Faculty of Health Sciences, University of Cape Town, Cape Town, South Africa; Medical Biotechnology & Immunotherapy Research Unit, Institute of Infectious Disease and Molecular Medicine (IDM), Faculty of Health Sciences, University of Cape Town, Cape Town, South Africa; South African Research Chair in Cancer Biotechnology, Department of Integrative Biomedical Sciences, Faculty of Health Sciences, University of Cape Town, Cape Town, South Africa; Medical Biotechnology & Immunotherapy Research Unit, Institute of Infectious Disease and Molecular Medicine (IDM), Faculty of Health Sciences, University of Cape Town, Cape Town, South Africa; Department of Molecular Pharmacology, Albert Einstein College of Medicine, Bronx, NY 10461, USA; South African Research Chair in Cancer Biotechnology, Department of Integrative Biomedical Sciences, Faculty of Health Sciences, University of Cape Town, Cape Town, South Africa; Medical Biotechnology & Immunotherapy Research Unit, Institute of Infectious Disease and Molecular Medicine (IDM), Faculty of Health Sciences, University of Cape Town, Cape Town, South Africa; South African Research Chair in Cancer Biotechnology, Department of Integrative Biomedical Sciences, Faculty of Health Sciences, University of Cape Town, Cape Town, South Africa; Medical Biotechnology & Immunotherapy Research Unit, Institute of Infectious Disease and Molecular Medicine (IDM), Faculty of Health Sciences, University of Cape Town, Cape Town, South Africa; Division of Immunology, Department of Pathology, Faculty of Health Sciences, University of Cape Town, Cape Town, South Africa; Division of Immunology, Department of Pathology, Faculty of Health Sciences, University of Cape Town, Cape Town, South Africa; Division of Immunology, Department of Pathology, Faculty of Health Sciences, University of Cape Town, Cape Town, South Africa; International Centre for Genetic Engineering and Biotechnology (ICGEB) and Institute of Infectious Diseases and Molecular Medicine (IDM), Division of Immunology, Faculty of Health Sciences, University of Cape Town, South Africa; Wellcome Centre for Infectious Diseases Research in Africa (CIDRI-Africa), Institute of Infectious Diseases and Molecular Medicine (IDM), Faculty of Health Sciences, University of Cape Town, South Africa; Department of Chemistry, Faculty of Sciences, University of Cape Town, Cape Town, South Africa; Department of Chemistry, Faculty of Sciences, University of Cape Town, Cape Town, South Africa; Department of Chemistry, Faculty of Sciences, University of Cape Town, Cape Town, South Africa; South African Research Chair in Cancer Biotechnology, Department of Integrative Biomedical Sciences, Faculty of Health Sciences, University of Cape Town, Cape Town, South Africa; Medical Biotechnology & Immunotherapy Research Unit, Institute of Infectious Disease and Molecular Medicine (IDM), Faculty of Health Sciences, University of Cape Town, Cape Town, South Africa

**Keywords:** Der p 1, house dust mite, Allergen-toxin, allergen-drug conjugate, Auristatin F and Pseudomonas exotoxin A

## Abstract

**Introduction:**

Current treatments for asthma help to alleviate clinical symptoms but do not cure the disease. In this study, we explored a novel therapeutic approach for the treatment of house dust mite allergen Der p 1induced asthma by aiming to eliminate specific population of B-cells involved in memory IgE response to Der p 1.

**Materials and Methods:**

To achieve this aim, we developed and evaluated two different proDer p 1-based fusion proteins; an allergen-toxin (proDer p 1-ETA) and an allergen-drug conjugate (ADC) (proDer p 1-SNAP-AURIF) against Der p 1 reactive hybridomas as an *in vitro* model for Der p 1 reactive human B-cells. The strategy involved the use of proDer p 1 allergen as a cell-specific ligand to selectively deliver the bacterial protein toxin Pseudomonas exotoxin A (ETA) or the synthetic small molecule toxin Auristatin F (AURIF) into the cytosol of Der p 1 reactive cells for highly efficient cell killing.

**Results:**

As such, we demonstrated recombinant proDer p 1 fusion proteins were selectively bound by Der p 1 reactive hybridomas as well as primary IgG1^+^ B-cells from HDM-sensitized mice. The therapeutic potential of proDer p 1-ETAʹ and proDer p 1-SNAP-AURIF was confirmed by their selective cytotoxic activities on Der p 1 reactive hybridoma cells. The allergen-toxin demonstrated superior cytotoxic activity, with IC_50_ values in the single digit nanomolar value, compared to the ADC.

**Discussions:**

Altogether, the proof-of-concept experiments in this study provide a promising approach for the treatment of patients with house dust mite-driven allergic asthma.

## Graphical Abstract

**Graphical Abstract:**
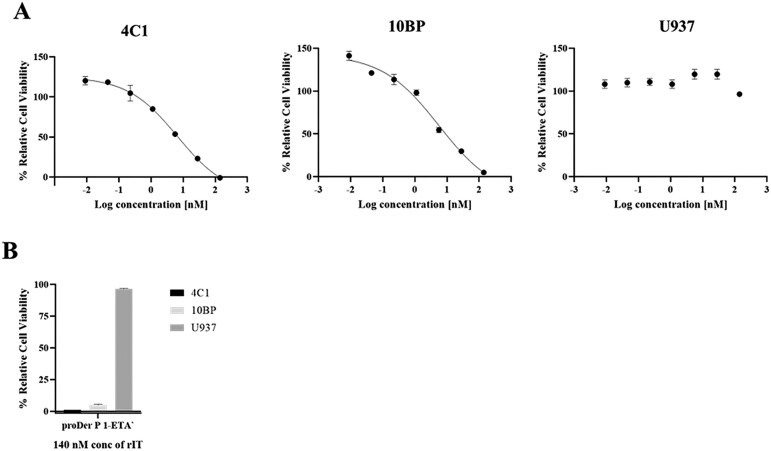

## Introduction

The prevalence of asthma has increased in most developed countries over the last decade [1]. By the year 2025, asthma is expected to affect about 400 million people worldwide [2]. In most countries, asthma compromises the patient’s quality of life and may result in mortality in severe cases [2]. Etiologically, asthma can be caused by many risk factors including genetic (activation of susceptibility genes), environmental (air borne pollutants, pollens, exercise, etc.), and host factors (obesity, stress, infections, allergen sensitization, etc.) [3, 4]. While clinical advances have been made in the treatment and management of asthma, there is currently no curative drug available for its treatment [5]. For more than 40 years, traditional therapies like β2-agonists and inhaled corticosteroids (ICS) have been used clinically to relieve patient symptoms [6, 7]. Unfortunately, these agents have short-term effects and do not cure the disease. Likewise, biological interventions in asthma are mostly based on monoclonal antibodies that neutralize the function of allergen-reactive IgE antibodies. Though these approaches showed some success when matched to the appropriate patient group, blocking the functions of circulating IgEs is unlikely to provide a cure for asthma since the primary source of the IgEs is not eliminated [8]. To maintain clinical benefits, patients are required to stay on anti-IgE medication for the rest of their lives, often with an impaired quality of life, a higher risk of adverse effect, asthma-induced hospitalization, or death.

In this study, we developed two targeted therapies (an allergen-toxin and an allergen-drug conjugate (ADC)) to eliminate all primary source of circulating IgE and by so doing provide a curative treatment for allergic asthma. Recent studies have shown that memory IgE responses are mediated by long-lived IgE producing plasma cells that can be directly reactivated by an allergen or by IgG1^+^ memory B-cells that can sequentially switch to IgE^+^ plasma cells upon allergen encounter [9]. Essentially, both memory responses are capable of producing the high-affinity IgE antibodies required for the activation of mast cells and basophils during a subsequent allergen exposure [9]. To this end, we identified both long-lived plasma cells and memory IgG1^+^ B-cells as potential therapeutic targets for allergen-based targeted therapy for the treatment of IgE-mediated chronic inflammation in allergic patients.

Technological-wise, an allergen-based targeted therapy will allow the selective elimination of allergen-reactive B-cell populations without compromising non-reactive B-cell populations. Of note, the success of targeted therapy in oncology has resulted in agents like Brentuximab vedotin and Moxetumomab pasudotox approved by the US Food and Drug Administration for the treatment of Hodgkin lymphoma and Relapsed/Refractory hairy cell leukemia respectively [10, 11]. The allergen-toxin and ADC developed in this study follows the format of an antibody based immunoconjugate. The cell binding domain consists of a recombinant inactive allergen (proDer p 1), whereas the effector domain is made up of the toxin (*Pseudomonas aeruninosa* exotoxin A (ETAʹ)) or the SNAP-tag protein labelled with benzylguanine (BG) modified mono methyl Auristatin F (AURIF).

These types of allergen-based drugs were first described about two decades ago [12, 13]. Lee and colleagues demonstrated the genetic fusion of ovalbumin and diphtheria toxin (OVA-DT) and its use to protect mice from ovalbumin induced anaphylactic shock. This proof-of-concept study showed that application of OVA-DT to OVA-sensitized mice destroyed OVA-specific IgE expressing B-cells and OVA-specific mast cells, and in addition, prevented systemic anaphylactic shock following OVA challenge post-treatment [12]. Proby *et al.* also reported the preparation of fusion proteins composed of desmoglein 3 fused to ETA and their use for the recognition and selective elimination of autoantigen-specific B-cells in a preliminary autoimmune diseases model of pemphigus vulgaris using immunized mice [13]. Furthermore, this principle was confirmed in *ex vivo* studies of Stöcker *et al.* demonstrating the therapeutic potency of such allergen-toxins to selectively eliminate Phl p 5b-reactive B-cells [14]. While these earlier studies demonstrated the first preclinical evidence for elimination of cells responsible for allergic reactions, they were eventually not followed up into clinical studies as the according indications are not representing life threatening diseases.

House dust mites (HDM) are commonly found in household items such as beds, carpets, and soft furniture and are known as producers of different (aero)allergens [15]. Globally, HDM derived allergens remain one of the most clinically relevant allergens [16]. People with HDM allergy often respond to proteins and endotoxins derived from the body and faeces of HDM. HDM produce several allergens; according to the WHO/IUIS database (http://allergen.org/) about 71 allergens have been identified, and more are still being studied. The HDM allergens classification are based on biochemical composition, homology, and molecular weight [17], the most dominant and clinically relevant of this group are the group 1 (Der p 1, Der f 1), group 2 (Der p 2, Der f 2) and group 23 (Der p 23) allergens. Group 1 allergens are excreted by the *Dermatophagoides pteronyssinus and Dermatophagoides farina* species [18, 19]. Structurally, Der p 1 (25-kDa) is synthesized as an inactive protein known as proDer p 1. This immature protein is composed of a catalytic domain of 222 residues, led by an N-terminal propeptide of 80 amino acids. This prosequence blocks the accessibility of the protease active site, and also covers some IgE epitopes of the mature allergen [20]. This pro-region is autocatalytically cleaved (under acidic conditions) upon enzyme maturation and exhibits papain-like cysteine proteases activity [21]. More than 80% of house dust mite allergic patients exhibit elevated serum anti-Der p 1 IgE titres [22, 23]. Furthermore, HDM allergen sensitization is estimated to affect 65 to 130 million people worldwide, and up to 50% of asthmatic patients [24]. In this study, two allergen-based therapeutics (an allergen-toxin proDer p 1-ETAʹ and an allergen-drug conjugate; proDer p 1-SNAP-AURIF) were prepared and evaluated for the treatment of house dust mite (HDM) allergen Der p 1 induced asthma.

## Methods

### Bacterial strains, Der p 1 allergen, and cloning

*Escherichia coli* strain DH5α (*supE44 ΔlacU169 (F80 lacZΔM15) hsdR17 recA1 endA1gyrA96 thi-1 relA1*) was used for molecular cloning and plasmid propagation, while the proDer p 1-ETAʹ and proDer p 1-SNAP fusion proteins were produced in E. coli strain BL21(λDE3) (*F- ompT hsdSB (rB- mB-) gal dcm- lon*). The complete Der p 1 nucleotide sequence was obtained from the National Center for Biotechnology Information protein database (accession no. ACG58378.1). The protein sequence was reverse translated into a DNA sequence and its 5ʹ and 3ʹ ends modified with *SfiI* and *NotI* restriction enzyme cutting sites before being synthesized commercially. All standard cloning procedures were performed as previously described [25].

### Expression and purification of allergen fusion proteins

Expression of recombinant proDer p 1-ETAʹ and proDer p 1-SNAP was achieved by employing the stress expression protocol previously described [1] for recombinant immunotoxin production in *E. coli*. Briefly, BL21 (λDE3) cells bearing the bacteria expression plasmid (pMT-H22(scFv)-ETAʹ or pMT-Der p 1-SNAP) were grown at 26°C until an optical density of OD_600_ nm of 1.6 was reached. At this OD, the cultures were supplemented with 0.5 M sorbitol, 4% NaCl, and 40 mM glycine-betaine monohydrate and cultured for 30 min. Protein expression was induced by the addition of 1 mM isopropyl β-d-1-thiogalactopyranoside (IPTG) and the cells cultured for 16 h with shaking at 180 rpm. For purification, the expressed fusion proteins were first purified by immobilized metal affinity chromatography (IMAC) on an ÄKTA Explorer system (GE, Healthcare) followed by size exclusion chromatography and analysed via SDS-PAGE and western blot.

### Cell culture

Der p 1 reactive hybridoma cell lines 4C1 and 10BP (Indoor Biotechnologies, USA) were cultured in complete DMEM medium (Gibco) supplemented with 10% (vol/vol) heat-inactivated fetal calf serum (FCS), 50 μg/ml penicillin, 100 μg/ml streptomycin. The cells were cultured at 37°C in a 5% CO_2_ air atmosphere.

### Der p 1 induced allergic asthma in mice

Balb/C Mice were housed in independently ventilated cages under specific pathogen-free conditions at the University of Cape Town Animal Facility. All mice were used at eight to 10 weeks of age and animal procedures were performed according to strict recommendation by the South African Veterinary Council and were approved by the University of Cape Town Animal Ethics Committee (Reference number 018/013).

Mice were sensitized while under anaesthesia (ketamine 80 mg/kg (Anaket-V; Centaur Labs) and xylazine 16 mg/kg (Rompun; Bayer) intratracheally on day 0 with 1 μg of HDM extract from *Dermatophagoides pteronyssinus* (Greer Laboratories, Lenoir, NC) resuspended in 50 μl PBS (Sigma-Aldrich, St Louis, MO) and intranasally challenged with 3 μg of HDM in 50 μl of PBS on days 8, 9, 10, 11, and 12 as previously described [26, 27]. PBS (50 μl) was administered as negative control. After the procedure, mice were euthanized.

### Isolation of mouse B-lymphocytes

Lungs (both lobes) and lymph nodes (mediastinal lymph nodes) from mice were homogenized and single-cell suspensions prepared in RPMI media (Gibco) by passing them through a 100-μm strainer. To obtain single-cell suspensions from lung tissues, the lobes of the lungs were digested for 1 hr at 37°C in RPMI media containing 13 mg/ml DNase I (Roche, Switzerland) and 50 U/ml collagenase IV (Gibco) and passed through a 70-μm strainer. Single cells were then blocked with 24G2 for 30 min at 4°C, followed by surface staining with fluorophore-conjugated FITC-B220 and phycoerythrobilin (PE)-CD19 for 30 min at 4°C in the dark. A dead cell exclusion dye (7AAD) was added before sorting on BD FACS Aria I to at least 96% purity.

### Flow cytometry and enzyme-linked immunosorbent assay

The ability of proDer p 1 fusion proteins to bind Der p 1 reactive hybridoma cells, or B-cells derived from HDM-sensitized mice was accessed by flow cytometry. Briefly, 1 × 10^6^ cells were suspended and incubated with 20 μg of either Alexa 647 labelled proDer p 1-ETAʹ or proDer p 1-SNAP-tag fusion protein in FACS buffer (PBS pH 7.4, 2 mM EDTA, 3 % (v/v) FCS) for 60 min on ice. Afterward, cells were washed twice in FACS buffer and analysed on a flow cytometer (BD Biosciences). An enzyme-linked immunosorbent assay (ELISA) was performed to determine the binding of IgE and IgG specific to Der p 1 as previously described [28].

### Internalization analysis by confocal microscopy

The ability of Der p 1 reactive membrane antibodies to internalize proDer p 1 fusion proteins after binding to Der p 1 reactive hybridoma cell lines was confirmed by confocal microscopy. Briefly, 96-well flat bottom microscopy plates (Ibidi, Germany) were plasma-treated with fibronectin (Thermo fisher scientific) to promote cell attachment. About 5 × 10^5^ cells (4C1/ 10BP) were plated and incubated with complete medium overnight. After 24 h, cells were washed with sterile 1× PBS (pH 7.4) before staining with 20 μg of Alexa 647 labelled proDer p 1-SNAP or proDer p 1-ETAʹ fusion proteins for 1 h. In some instances, labelling of cells was carried out on ice (to reduce cell metabolism and by so doing prevent internalization of fusion proteins). After staining, the cells were washed twice with 1× PBS (pH 7.4) and counterstained with the nuclear stain; DAPI. Next, cells were washed and fixed with 2% paraformaldehyde. Images were taken using the LSM 880 Airyscan confocal microscope (Zeiss) at 40× magnification.

### Cell viability assay

The therapeutic potential of proDer p 1-ETAʹ and proDer p 1-SNAP-AURIF for the selective elimination of Der p 1 reactive hybridoma cells was compared by a cell viability assay (XTT assay) using the cell proliferation kit (Sigma-Aldrich, USA). Briefly, 5 × 10^3^ cells were seeded/well overnight in 96-well plates in complete medium (DMEM medium supplemented with 10% (v/v) FBS and 1% penicillin streptomycin) at 37°C and 5% CO_2_. After 24 h, the cells were treated with decreasing concentration of the allergen-rIT or allergen-drug conjugate for 48 h after which the XTT reagent was added to each well and incubated for another 4 h. Zeocin^®^ (100 μg/ml) was used as a control to achieve 100% cell killing. The readout was conducted by measuring absorbance of the reduced XTT reagent at 450 nm with a reference of 655 nm on a spectrophotometer (Bio-Rad).

## Results

### Structural characterization of proDer p 1-ETAʹ and proDer p 1-SNAP expressed in bacteria

**Figure 1.**
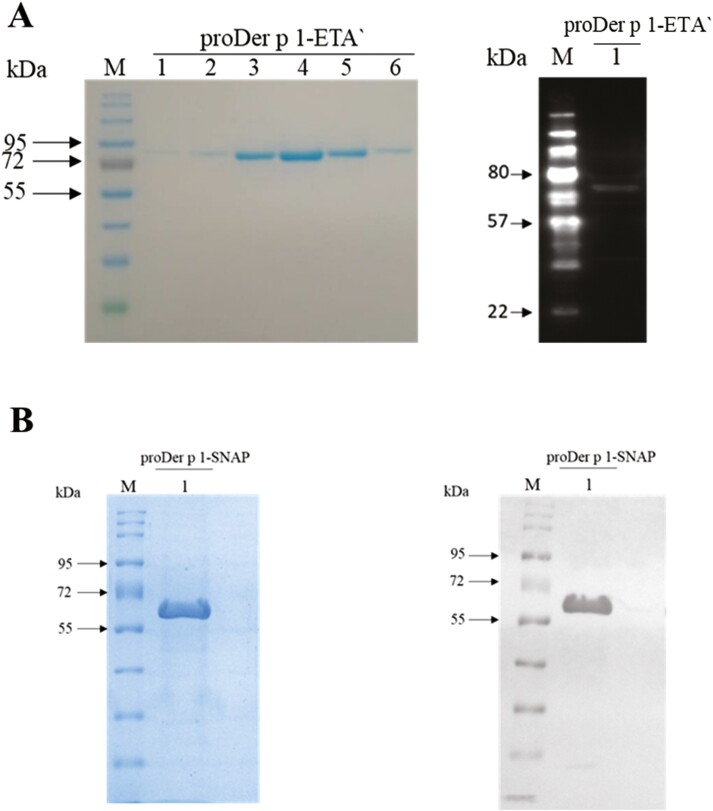
Production and structural characterization of proDer p 1-ETAʹ and proDer p 1-SNAP. Representative 10% SDS-PAGE gels and western blots of full-length proDer p 1-ETAʹ (A) and proDer p 1-SNAP (B) after size exclusion chromatography (SEC). Lanes 1–6 on figure 1A (left panel) are representative of protein fractions after SEC. Western blots were performed using an anti-His_tag_ antibody for the specific detection of a histidine tag at the N-terminus of the fusion proteins. M = Protein Ladder.

Inactive proDer p 1 (proteolytically inactive) allergen fusion proteins, proDer p 1-ETAʹ (79.7 kDa) and proDer p 1-SNAP (57 kDa), were expressed in bacteria using the osmotic stress expression protocol described by Barth et al. [29]. The recombinant proteins were purified from the periplasmic space using a combination of Ni-NTA IMAC and size exclusion chromatography. To confirm the successful expression of the allergen fusion proteins in *E. coli*, the purified proteins were characterized by resolving on a 10% SDS-PAGE gel and detected by western blot using an anti-his-tag antibody. The osmotic stress expression protocol resulted in successful expression of full-length allergen fusion proteins (Figure 1). The average final concentrations of purified protein from 1 l of bacteria culture were 2 mg/l for proDer p 1-ETAʹ and 3.5 mg/l for proDer p 1-SNAP.

### Binding of proDer p 1-SNAP to Der p 1 reactive hybridoma cells

**Figure 2.**
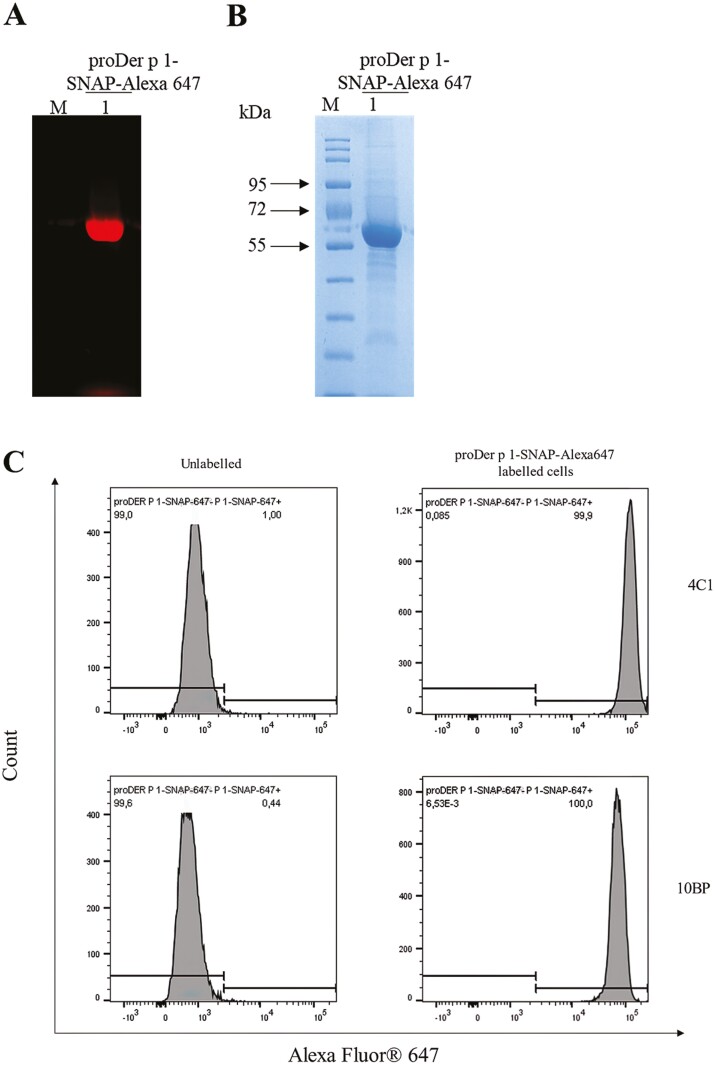
Flow cytometry studies of proDer p 1-SNAP binding to Der p 1 reactive hybridoma cell lines. About 5 µM concentration of proDer p 1-SNAP fusion protein was labelled with two-fold molar excess of SNAP-Surface™ Alexa Fluor^®^ 647 (A and B). Thereafter, successful labelling of the fusion protein was confirmed by running the conjugation product on an SDS-PAGE gel. (C) The binding activities of proDer p 1-SNAP was thereafter investigated by staining Der p 1 reactive hybridoma cell lines (4C1, 10BP) with 20 µg of Alexa Fluor^®^ 647 labelled proDer p 1-SNAP fusion protein for 1 h. Afterwards, was data acquired on the BD LSR-II flow cytometer and analysed on the Flowjo v10.7.2 software.

The ability of bacterially expressed recombinant proDer p 1 fusion protein to bind to Der p 1 reactive hybridoma cell lines (4C1 and 10BP) was evaluated by cell binding studies using flow cytometry. For this, the proDer p 1-SNAP fusion protein was used. Before commencing the binding studies, the proDer p 1-SNAP fusion protein was labelled with a benzylguanine (BG)-modified fluorophore, SNAP-Surface™ Alexa Fluor^®^ 647 (New England Biolabs), as described by the manufacturer. The successful labelling of the fusion protein was confirmed by SDS-PAGE and visualized with the iBrightFL1000 imaging system (Figure 2A and B). Following incubation of the fusion protein with each hybridoma cell line the labelled cells were subsequently analysed on the flow cytometer. The proDer p 1-SNAP fusion protein demonstrated adequate binding affinity for the receptor on both 4C1 and 10BP Der p 1 reactive hybridoma cells (Figure 2C).

### Internalization studies of proDer p 1-ETAʹ and proDer p 1-SNAP allergen fusion proteins into Der p 1 reactive hybridomas

**Figure 3.**
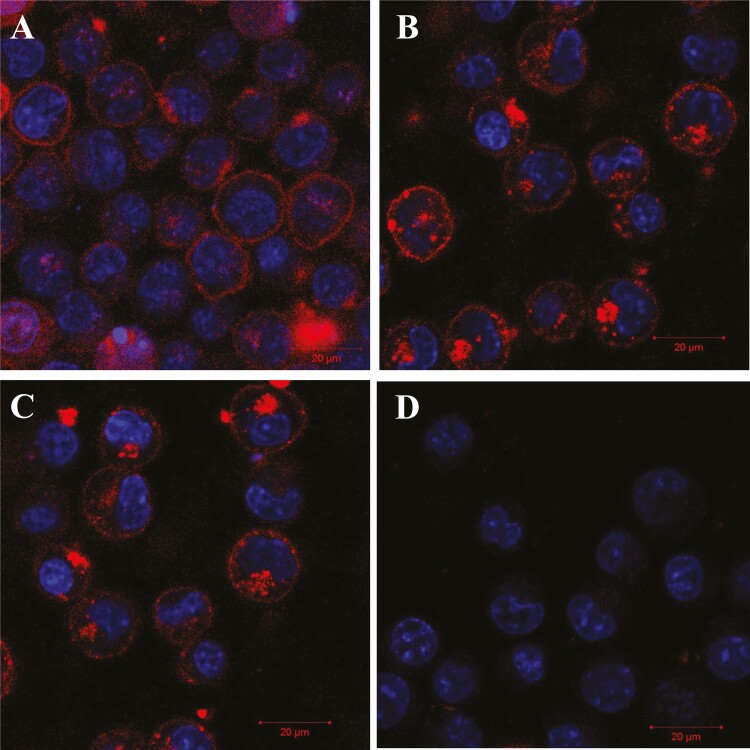
Binding and internalization kinetics of proDer p 1-SNAP and proDer p 1-ETAʹ fusion proteins. The binding and internalization activities of Alexa Fluor^®^ 647 labelled proDer p 1-SNAP and proDer p 1-ETAʹ was investigated by staining 4C1 Der p 1 reactive hybridoma cells with each fusion protein. Confocal microscopic analysis confirmed the ability of Der p 1 reactive cells to selectively bind and internalize the proDer p 1 fusion proteins. (A) 4C1 cells labelled on ice with proDer p 1-SNAP for 1 h, (B and C) 4C1 cells labelled for 1 h with proDer p 1-SNAP-Alexa Fluor^®^ 647 and proDer p 1-ETAʹ-Alexa Fluor^®^ 647 at 37°C, respectively, (D) 4C1 cells labelled with anti-CSPG4(scFv)-SNAP-Alexa Fluor^®^ 647 fusion protein. The images were acquired using the Zeiss LSM 880 Airy scan confocal microscope.

In addition to their specific binding abilities, the fusion proteins developed in this study need to be effectively internalized into their target cells in order to exert cytotoxic activity. Therefore, the specific binding and internalization kinetics of Alexa Fluor^®^ 647 labelled proDer p 1-SNAP and proDer p 1-ETAʹ was assayed by confocal microscopy. For this assay, the Der p 1 reactive hybridoma cell line; 4C1 was used for proof-of-concept establishment. At first, surface binding of proDer p 1-SNAP was accessed by labelling the 4C1 cells on ice. Upon confirmation of binding by successful membrane labelling (Figure 3A), the ability of Der p 1 reactive 4C1 cells to internalize both proDer p 1-SNAP and proDer p 1-ETAʹ was assayed by incubating the cells with each fusion protein for 1 h. Confocal microscopy analysis showed that fluorescent signals representative of the labelled fusion proteins was present on the cell surface, but mainly in vesicles in the cytosol in a pattern typical to receptor-mediated endocytosis (Figure 3B and C). As a negative control, an unrelated antibody fusion protein (anti-CSPG4(scFv)-SNAP) was also labelled with BG-modified Alexa 647. Incubation of 4C1 cells with anti-CSPG4(scFv)-SNAP-Alexa Fluor^®^ 647 fusion protein under the same experimental conditions resulted in no binding or internalization (Figure 3D), demonstrating antigen-specific internalization.

### proDer p 1-ETAʹ selectively kills Der p 1 reactive hybridoma cell lines

**Figure 4.**
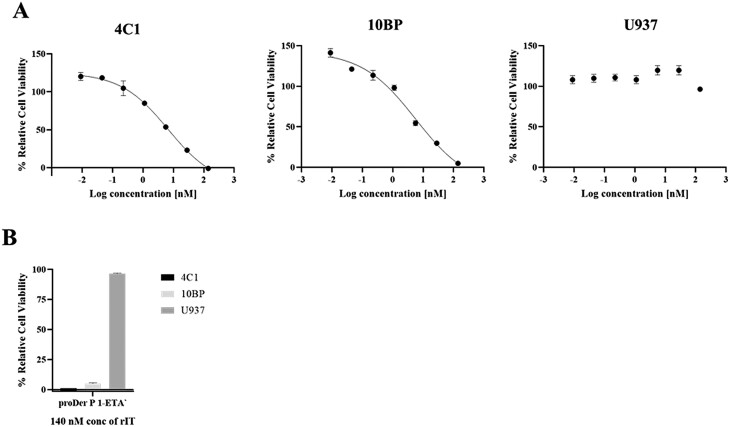
Cytotoxic activity of proDer p 1-ETAʹ immunotoxin on 4C1 and 10BP hybridoma cell lines. (A) Der p 1 reactive hybridoma cells (4C1 and 10BP) and U937 human monocytic cell line (negative control) were seeded in quadruplicate. After 24 h, the cells were treated with decreasing concentration of proDer p 1-ETAʹ allergen-toxin. After 48 h of treatment, the half-maximal inhibitory concentration (IC_50_) of proDer p 1-ETAʹ relative to untreated and zeocin (positive) controls was calculated using the GraphPad Prism V8 software. Dose-dependent decreasing viability of 4C1 and 10BP is shown as sigmoidal curve with an IC_50_ of 6.6 nM and 5.7 nM, respectively. (B) Cell viability of 4C1, 10BP and U937 cells after incubation with 140 nM of proDer p 1-ETAʹ.

The *in vitro* cytotoxic activity of proDer p 1-ETAʹ was investigated (XTT assay) following the successful confirmation of its binding and internalization activity by confocal microscopy. Briefly, decreasing doses of the proDer p 1-ETAʹ immunotoxin was incubated with the Der p 1 reactive hybridoma cell lines. The U937 human monocytic cell line was used as a negative control. The proDer p 1-ETAʹ immunotoxin was selectively toxic to both 4C1 and 10BP hybridoma cell lines and not to the U937 cells. Treatment with proDer p 1-ETAʹ resulted in a significant reduction in cell viability with an IC_50_ value of 6.6 nM and 5.7 nM for both 4C1 and 10BP, respectively. In contrast, the negative cell line was not affected by the highest immunotoxin concentration used (140 nM) (Figure 4A and B).

### *Ex vivo* binding analysis of proDer p 1 fusion proteins to B-lymphocytes

**Figure 5.**
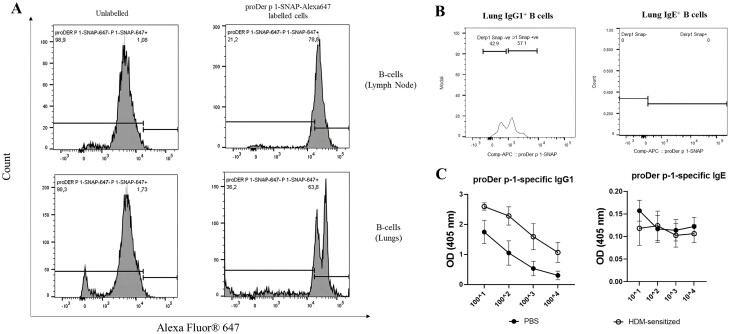
*Ex vivo* assessment of proDer p 1 fusion protein binding to B-lymphocytes isolated from HDM-sensitized mice. To demonstrate the therapeutic application of proDer p 1-based fusion proteins, CD19 and B220 sorted B-lymphocytes from HDM-sensitized mice were incubated with Alexa Fluor^®^ 647 labelled proDer p 1-SNAP fusion proteins for 1 h on ice before flow cytometric analysis. (A) Representative flow cytometric results showed that about 80% of B-cells from lymph nodes and 70% of B-cells from lungs were positive for proDerp 1-SNAP. proDer p 1-SNAP was labelled with benzylguanine-modified fluorophore (SNAP-Surface™ Alexa Fluor^®^ 647). (B) B-cells from HDM-sensitized mice were labelled with IgG1 and IgE specific antibodies. About 57% of sorted IgG1^+^ B-cells were reactive to proDer p 1-SNAP while no IgE positive B-cell population could be detected. (C) The ability of recombinant proDer p 1 to bind Der p 1 reactive antibodies from HDM-sensitized mice was demonstrated by antibody ELISA. proDer p 1-SNAP binds to IgG1 antibodies from serum of HDM-sensitized mice. All assays were repeated in two biological repeats.

To analyse the therapeutic potential of proDer p 1-based fusion proteins, the potency of proDer p 1-SNAP binding to Der p 1 reactive B-cells isolated from HDM-sensitized mice was examined *ex vivo*. A low dose treatment schedule with HDM extract was used to induce HDM allergic asthma in female Balb/c mice. Upon successful induction of allergic asthma (confirmed by lung function assay, data not shown), mice were euthanized and single-cell suspensions from the lungs and lymph nodes prepared using standard protocols. The sorted B-cells were immediately stained with Alexa Fluor^®^ 647 labelled proDer p 1-SNAP fusion protein. About 5 × 10^5^ B-cells (from lungs/lymph nodes) were incubated with Alexa Fluor^®^ 647 labelled proDer p 1-SNAP fusion protein on ice for 1 h. Flow cytometric analysis showed that approximately 80% and 70% of B-cells from lymph nodes and lungs were positive for proDer p 1-SNAP-Alexa Fluor^®^ 647 respectively (Figure 5A). To confirm the specific B-cell isotype binding to proDer p 1-SNAP, we labelled B-cells from the lungs of HDM-sensitized mice with IgG1 and IgE antibodies. As shown in Figure 5B, 57% of sorted IgG1^+^ B-cells were reactive to proDer p 1-SNAP. On the other hand, the availability of IgE^+^ proDer p 1 reactive B-cells could not be confirmed due to absence of IgE^+^ B-cell population during cell sorting (Figure 5B). In addition to flow cytometry data, the ability of proDer p 1-SNAP to bind antibodies from HDM-sensitized mice was confirmed by antibody ELISA. As shown in Figure 5C, IgG1 antibodies present in the serum of HDM-sensitized mice were reactive to proDer p 1 (Figure 5C). Altogether, these results confirm binding of proDer p 1 fusion proteins to IgG^+^ B-cells derived from HDM-sensitized mice, thus further confirming its therapeutic potential for future *in vivo* studies.

### Preparation of a novel ADC

**Figure 6.**
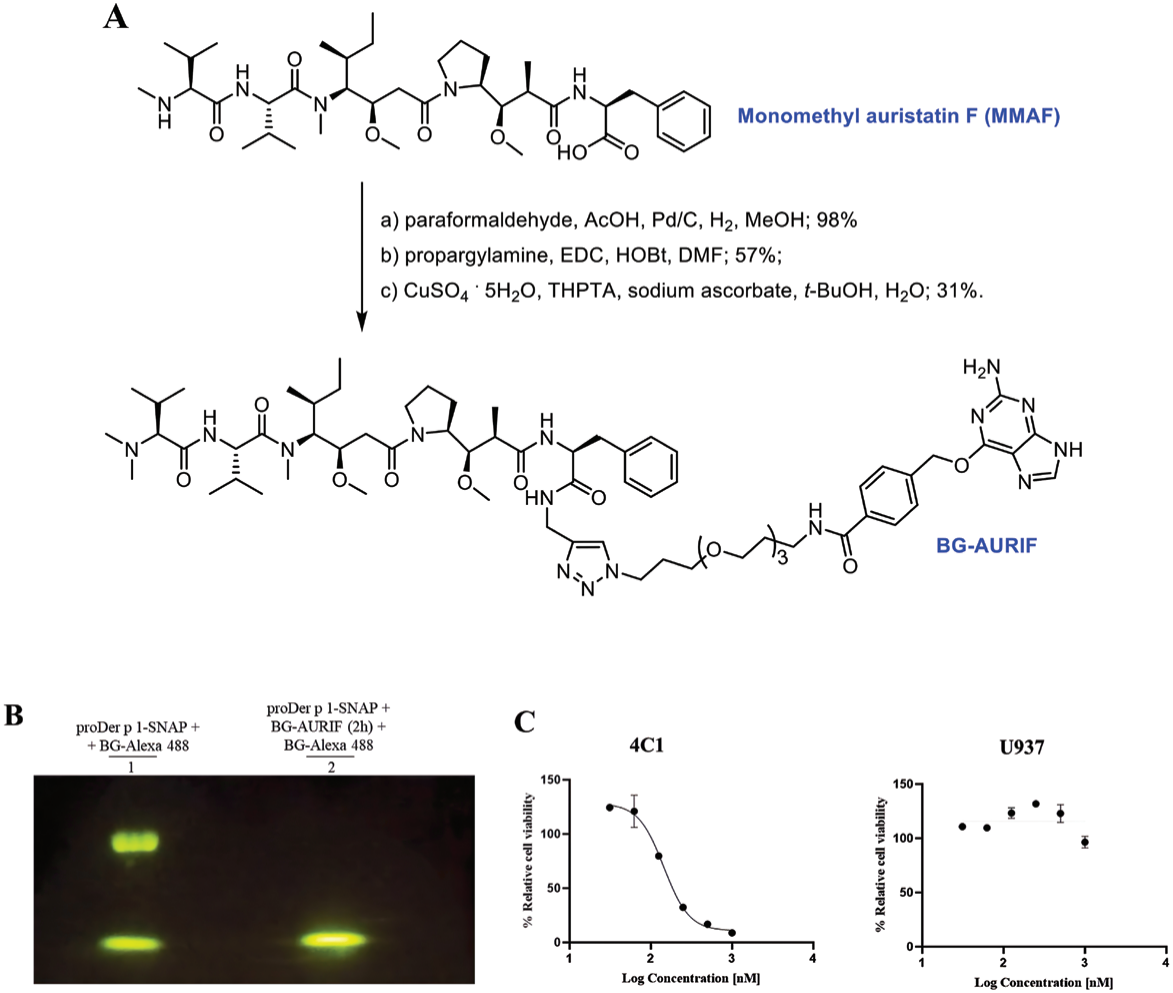
Development and cytotoxic activity of novel proDer p 1-SNAP allergen-drug conjugate. (A) Chemical modification of monomethyl auristatin F (MMAF) first using reductive amination at its N-terminus, conversion of the C-terminus to its N-propargylamide followed by click coupling (CuAAC) with an appropriate BG-linker-azide construct afforded BG-AURIF for a one step site-specific covalent conjugation to the proDer p 1-SNAP fusion protein. (B) Corresponding SDS-PAGE gel was used to confirm the conjugation efficiency of BG-AURIF to proDer P 1-SNAP. Lane 1 and 2 showed that prior labelling of proDer P 1-SNAP with 2-fold molar excess of BG-AURIF was enough to saturate the active site of the SNAP-tag molecule. (C) Der p 1 reactive hybridoma cells (4C1) and U937 human monocytic cell line (negative control) were seeded in quadruplicate and after 24 h cells were treated with decreasing concentration of proDer p 1-SNAP-AURIF in complete DMEM media. The half-maximal inhibitory concentration (IC_50_) of proDer p 1-SNAP-AURIF relative to untreated and zeocin controls was calculated using the GraphPad Prism V8 software. Dose-dependent decreasing viability of 4C1 is shown as sigmoidal curve with an IC_50_ of 144 nM.

The proDer p 1-SNAP fusion protein and cytotoxic tubulin inhibitor, monomethyl auristatin F (MMAF), was used to develop an allergen-drug conjugate for the selective killing of Der p 1 reactive cells. To allow a 1:1 stoichiometric conjugation to the SNAP-tag fusion protein, MMAF was chemically N-methylated and modified at the C-terminus with a linker-BG moiety containing a benzylguanine group for SNAP-tag conjugation. The resultant benzylguanine-linker-auristatin F construct was thereafter named BG-AURIF (Figure 6A). An original article describing the synthesis of BG-AURIF can be found under Huysamen, A. M. et al. (2023) in ACS Omega. Subsequently, proDer p 1-SNAP was labelled with a 2-fold molar excess of BG-AURIF in phosphate buffer at 37°C for 2 h. The labelled protein was thereafter purified using an Amicon^TM^ ultracentrifugation filter device (10 kDa MWCO) to remove residual/unbound BG-AURIF. To confirm the successful preparation of proDer p 1-SNAP-AURIF as a novel allergen-drug conjugate, the BG-AURIF labelled protein was subjected to a secondary conjugation with BG-Alexa 488 fluorophore and analysed on an SDS-PAGE. Failure of BG-Alexa 488 conjugation to the BG-AURIF labelled protein relative to control confirmed that prior conjugation of BG-AURIF to the proDer p 1-SNAP fusion protein was complete and successful (Figure 6B).

### Cytotoxic activities of proDer p 1-SNAP-AURIF

The XTT cell proliferation assay was used to investigate the cytotoxic activity of proDer p 1-SNAP-AURIF. For this assay, Der p 1 reactive hybridoma cells 4C1 were treated with decreasing doses of proDer p 1-SNAP-AURIF for 48 hr after which the viability of the cells was compared with media and zeocin-treated (positive) controls. Quantitative analysis of cell viability showed that proDer p 1-SNAP-AURIF was active against 4C1 cells (Figure 6C). The allergen-drug conjugate compromised cell viability in a dose-dependent manner with an IC_50_ value of 144 nM. The selectivity of proDer p 1-SNAP-AURIF for only Derp 1 reactive cells was demonstrated by using the CD64^+^ cell line, U937, as a negative control. Treatment of this cell line with a decreasing dose of proDer p 1-SNAP-AURIF showed no effect on cell viability.

## Discussion

In this study, we produced and evaluated two novel Der p 1-based fusion proteins for the selective elimination of populations of B-cells required for the generation of Der p 1 memory IgE responses. It is well acknowledged that allergen-reactive IgEs are responsible for both the immediate allergic response and late-phase chronic inflammatory response seen in patients [30]. In these patients, the synthesis of IgE is initiated by the interaction of inhaled allergen with antigen presenting cells that line the airway (e.g., dendritic cells) [31]. After migrating to the lymph nodes, antigen presenting cells present processed antigens to T helper cells in an interaction that leads to the activation of Th2 cells. Interleukins (IL-4 and IL-13) released by the activated Th2 cells provide the first signal to B-cells to switch from IgM or IgG to IgE antibody-producing B-cells [32]. When present, IgE antibodies briefly circulate in the blood before binding to cells with IgE receptors (e.g., mast cells and basophils) via the IgE constant region [33]. The presence of IgE on mast cells in the nasal or bronchial mucosa eventually makes an individual highly sensitive to allergens. In these individuals, a subsequent allergen exposure leads to mast cell activation due to intracellular signalling generated by physical crosslinking of allergen bound IgEs [34]. This results in the release of preformed proinflammatory mediators (such as histamine and leukotrienes) responsible for the immediate allergic reaction seen in patients. Although this early-phase reaction resolves within an hour, the released mast cell mediators also induce a prolonged late-phase reaction that results in the obstruction of airflow about 4–8 h later [30].

Interestingly, positive clinical results from the use of omalizumab have demonstrated that the effects of allergic asthma can be controlled by blocking the activities of IgE antibodies [35]. By preventing circulating IgEs from binding to mast cells and basophils, omalizumab has been reported to prevent/attenuate both early-phase and late-phase airway-obstructive response to allergens in patients [35]. Nonetheless, a major limitation with the use of omalizumab is that allergic patients using omalizumab need to stay on treatment for the rest of their life to maintain clinical benefit (Omalizumab is not curative). In the event where treatment is stopped, patients have reported the return of asthma symptoms to pre-treatment levels within a relatively short period of time [36]. In addition, omalizumab is a high-cost drug (about $15,000, to $44,000 USD per year depending on dosage), and not affordable in the long-term for the average patient [37]. Each administration of omalizumab also carries the risk of an anaphylactic shock and other side effects. Against this backdrop, the development of next-generation therapies for selectively eliminating B-cells providing the primary source of allergen-reactive IgEs seems plausible.

As described earlier, we evaluated two allergen-based targeted therapies for the selective elimination of Der p 1 specific long-lived plasma cells and memory IgG1^+^ B-cells. The allergen fusion proteins developed in this study consist of an inactive proDer p 1 allergen as the cell-targeting domain. Inactive proDer p 1 was used since proteolytically active mature Der p 1 is capable of degrading airway anti-protease lung defence mechanisms (such as α1-antitrypsin inhibitor or secretory leukocyte protease inhibitor), leading to enhanced tissue damage and innate immune activation [38, 39]. Firstly, the results from this study document for the first time the successful expression of soluble and functional recombinant proDer p 1-based fusion proteins in *E. coli*. It has been reported that the expression of recombinant Der p 1 in *E. coli* is difficult, often result in accumulation of improperly folded proteins in inclusion bodies. For this reason, many studies have reported strategies for proDer p 1 expression in *P. pastoris* [40]. The osmotic stress expression protocol described in this study allowed reproducible expression rates of about 2 mg/l and 3.5 mg/l of proDer p 1-ETAʹ and proDer p 1-SNAP from *E. coli* shaking cultures. These findings are similar to previous studies from Nachreiner *et al.*, and Klose *et al.*, who reported the recovery of more than 95% functional protein from using the same periplasmic osmotic stress expression protocol [41, 42]. Osmotic stress in the presence of compatible solutes during protein expression created an intracellular environment to support the correct folding of the expressed allergen-toxin [29].

By employing both flow cytometry and confocal microscopy, we were able to confirm the ability of recombinant proDer p 1 to bind and be internalized by transmembrane-bound antibodies on 10BP and 4C1 hybridoma cells. Though no information is available on the type of transmembrane antibodies present on the 10BP and 4C1 hybridoma cells, the antibodies secreted by both cells are of the IgG1 isotype and can be commercially sourced. To this end, we expect the binding and internalization of proDer p 1 fusion proteins to be mediated by mIgG1 on the 10BP and 4C1 hybridoma cell lines. Furthermore, the ability of memory IgG1^+^ B-cells from HDM sensitize mice to bind our recombinant proDer p 1 fusion proteins was confirmed by flow cytometry. B-cells from the lung tissue of HDM-sensitized mice were labelled with a cocktail of proDer p 1-SNAP and murine IgG1 and IgE specific antibodies. The results showed that about 60% of the IgG1^+^ B-cells were reactive and bound to proDer p 1-SNAP after 60 mins of incubation. Notably, the high binding affinity demonstrated by recombinant proDer p 1-SNAP fusion proteins for IgG1^+^ B-cells is important for an enriched intracellular accumulation and biological activity in target memory IgG1^+^ B-cells. Though this study also aimed to target long-lived IgE^+^ plasma B-cells, we could not demonstrate binding of proDer p 1-SNAP to IgE^+^ B-cells or IgE antibodies from HDM-sensitized mice. Essentially, this can be due to the fact that IgE^+^ B-cells are rare and difficult to detect *ex vivo* [43]. Serum IgE are also known to have a short half-life of 12 h [44] making it difficult to detect in an ELISA. Nevertheless, it is important to confirm that the conformational epitopes of Der p 1 are present on our *E. coli* expressed recombinant fusion proteins. Theoretically, it would be expected that Der p 1 reactive IgE would bind to both proDer p 1-ETAʹ and proDer p 1-SNAP since they have been described to compete with the same epitope as Der p 1 reactive IgG [28]. In short, the ability of recombinant proDer p 1-SNAP to bind primary B-cells from HDM-sensitized mice strengthens the hypothesis that recombinant inactive proDer p 1 protein can be used as a targeting ligand for targeted therapies against Der p 1 induced asthma. The IC_50_ values were used to compare the cytotoxic potential of proDer p 1-ETAʹ and proDer p 1-SNAP-AURIF towards the Der p 1 reactive hybridomas. proDer p 1-ETAʹ demonstrated specific and potent cytotoxicity towards 4C1 and 10BP cells (IC_50_ value of 6.6 nM and 5.7 nM respectively). Notably, these IC_50_ values are relatively comparable to those obtained in cancer immunotherapy: the anti-EGFR(scFv) immunotoxins; scFv1711-ETAʹ and scFv2112-ETAʹ developed by Niesen *et al.*, for the treatment of solid tumours demonstrated IC_50_ values ranging from 4 pM to 0.46 nM [45]. In a recent study, Rodrigo *et al.* also reported on the development and evaluation of a Der p 1 allergen-toxin based on the ribotoxin: α-sarcin [46]. This construct named proDerp1αS was expressed in yeast *P. pastoris* and proof of principle evaluated on human basophil-like cells (humRBL-2H3) sensitized with sera from Der p 1 allergic patients. The authors showed that proDerp1αS could bind, activate, and kill serum sensitized humRBL-2H3 cells. The authors showed that the cytotoxicity of proDerp1αS on humRBL-2H3 was selective and dependent on the presence of Der p 1-specific IgE on the cell surface. An IC_50_ of greater than 100 nM was however, reported by the authors for proDerp1αS against the humRBL-2H3 cells, with about 1 μM of proDerp1αS needed to achieve 75–80% decrease in cell viability. In comparison, the single digit nanomolar IC_50_ values obtained in this study for proDer p 1-ETAʹ against 4C1 and 10BP cells might be indicative of the toxin (ETAʹ) moiety being more potent than the α-sarcin in proDerp1αS. It also remains to be confirmed if the stress expression protocol described in this study might be more suitable for the expression of highly functional recombinant proDer p 1 fusion proteins in comparison to *P. pastoris*. Altogether, the results from the use of both proDer p 1-ETAʹ and proDerp1αS support earlier data from Lee *et al.*, Stöcker *et al.*, and others on the development of allergen-toxin for targeted asthma therapy [12–14]. Finally, the allergen-drug conjugate (proDer p 1-SNAP-AURIF) developed in this study also demonstrated selective cytotoxic activity towards 4C1 hybridoma cells in a dose-dependent manner. Though the cytotoxic activity of the allergen-toxin was significantly higher than the allergen-drug conjugate (IC_50_ 6.6 nM vs 144 nM against 4C1 cells), these result documents the first allergen-drug conjugate developed for the treatment of allergic asthma. It confirms a potential clinical value for allergen-drug conjugates when developed for asthma therapy.

In conclusion, the goal of the present work was to demonstrate a proof-of-concept for the selective elimination of diseased promoting B-cells in human asthma as a novel strategy for asthma drug therapy. This study successfully documented two allergen-based targeted therapies as an allergen-toxin and an allergen-drug conjugate for the selective elimination of Der p 1 reactive hybridoma cells (a comparative *in vitro* model for Der p 1 reactive B-cells in patients). Though other immune effector cells like mast cells and basophils are implicated in the pathology of allergic asthma, the absence of serum allergen-reactive IgEs (by the killing of memory IgG1^+^ B-cells and long-lived IgE^+^ plasma cells) would prevent crosslinking of FcεRI bound IgE and activation of these immune cells. It is however important to note that not all patients might benefit from an allergen-based targeted therapy. A study by Cruz *et al.*, showed that 36 out of 98 asthma patients who had tested positive for an immediate hypersensitivity skin test to HDM had circulating IgG antibodies against Der p 1 in their serum samples [47]. In such patients, these antibodies can bind and neutralized recombinant proDer p 1 fusion proteins and prevent them from reaching their target cells and by so doing compromise the expected clinical outcome. Going forward, we are currently busy with further improving the purification of our protein preparation including proper endotoxin removal and are aiming to use the results of this publication to get the funding needed to run the preclinical animal model available here in Cape Town. Nevertheless, the preclinical findings from this study demonstrate that both proDer p 1-ETAʹ and proDer p 1-SNAP-AURIF allergen fusion proteins merit further evaluation for the treatment of allergic asthma.

## Data Availability

The authors confirm that the data supporting the findings of this study are available within the article

## Abbreviations

ADC: Allergen-drug conjugate
AURIF: Auristatin F
BG: Benzylguanine
ELISA: Enzyme-linked immunosorbent assay
ETA: Pseudomonas exotoxin A
FCS: Foetal calf serum
FDA: US Food and Drug Administration
HCL: Hairy cell leukemia
HDM: House dust mites
HL: Hodgkin lymphoma
ICS: Inhaled corticosteroids
IHST: Immediate hypersensitivity skin test
IPTG: Isopropyl β-d-1-thiogalactopyranoside
MMAF: Monomethyl auristatin F
NCBI: National Center for Biotechnology Information
OVA-DT: Ovalbumin and diphtheria toxin
PE: Phycoerythrobilin
